# Riparian Areas and Fine‐Scale Forest Cover and Structure Drive Occupancy Patterns of Sympatric Mustelids

**DOI:** 10.1002/ece3.71370

**Published:** 2025-05-07

**Authors:** Lauren Wheelhouse, Heather Bryan, Shannon Crowley, Chris Johnson, Dexter Hodder

**Affiliations:** ^1^ John Prince Research Forest University of Northern British Columbia Prince George British Columbia Canada; ^2^ Natural Resources and Environmental Studies Graduate Program University of Northern British Columbia Prince George British Columbia Canada; ^3^ Department of Ecosystem Science and Management University of Northern British Columbia Prince George British Columbia Canada

**Keywords:** ecology, habitat, mesocarnivores, occupancy, riparian

## Abstract

Boreal and subboreal forests of western North America support diverse mesocarnivore populations with overlapping spatial, temporal, and dietary niches. However, interactions among these species and the factors influencing their co‐occurrence—such as population cycles, landscape changes, harvest mortality, and resource availability—are not well documented. We used 5 years (2015, 2016, 2020, 2021, and 2022) of data from remote cameras and fine‐scale habitat data from Light Detection and Ranging to assess patterns in the spatial co‐occurrence of short‐tailed weasel ( *Mustela erminea* ), American mink (*Neogale vison*), American marten ( *Martes americana* ), and fisher (*Pekannia pennanti*) in central British Columbia, Canada. We used multispecies occupancy models to test hypotheses about the relationships between mesocarnivore co‐occurrence and habitat. Mesocarnivores were more likely to co‐occur at sites closer to riparian zones and at sites with greater complexity of vertical forest structure. Short‐tailed weasel, however, did not co‐occur with other mustelids in riparian zones. Importantly, we found that habitat covariates associated with co‐occurrence were relatively similar over time despite changes in the abundance of predators and prey. Our findings highlight the importance of retaining riparian habitats and forest complexity as part of forest harvesting practices to promote species co‐occurrence.

## Abstract 2

We observed the co‐occurrence of mesocarnivores in Central British Columbia using 5 years of camera trap data and fine‐scale habitat measurements using Light Detection and Ranging. We found that forest structure and riparian habitats provided suitable habitats for mink, marten, and fisher to co‐occur within. Our findings highlight the importance of retaining forest structure during forest harvesting operations.

## 1 Introduction

Sympatric species interact in complex ways and may compete for shared resources such as food and space (Whittaker and Levin 1975). One mechanism that facilitates species co‐occurrence is spatial niche partitioning, which can occur when species occupy different sites or when species use different microhabitats within a site (Tilman and Kareiva 1997). Patterns in spatial niche partitioning are influenced by community dynamics such as the relative abundance of predators, competitors, and food resources (Murray et al. 2023). Habitat characteristics that influence species interactions and resource availability also have strong effects on spatial niche partitioning and, by extension, patterns in species co‐occurrence (Zhong et al. 2016). Understanding variation in species co‐occurrence can provide insight into mechanisms that allow species to share space.

Forest harvesting can influence interspecific interactions through changes in population dynamics (Evans and Mortelliti 2022). For example, forest harvesting may increase the abundance of species that use early seral stands (Parsons et al. 2020) and decrease the abundance of species that depend on more mature seral stands, such as marten (Fuller and Harrison 2005). Crowley et al. (n.d.) studied Canada lynx habitat use relative to forest harvesting, revealing cycle‐dependent patterns. Changes in abundance due to forest harvesting can interact with ecological cycles and influence population dynamics (Ferron et al. 1998).

Riparian areas are habitats that are typically retained during forest harvesting because of regulations that protect forests adjacent to waterbodies. Riparian is a general term describing a diverse group of habitats, including shorelines of lakes, wetlands, rivers, and streams (Verry et al. 2004). Riparian areas are often structurally complex due to gaps in the canopy and an abundance of water (Verry et al. 2004); consequently, species diversity may be higher in areas closer to riparian features compared with those farther away (Kremsater and Bunnell 1999). Although riparian management has historically focused on the protection of aquatic species, there has been a shift to explore the importance of riparian areas for terrestrial species (Li et al. 2024). When mature forests are harvested, many animal species may retreat to the nearest suitable habitat, including riparian areas (Courtois et al. 2008). That includes riparian forests that are typically retained as part of efforts to maintain fish populations. For example, American marten ( *Martes americana* ) is a species that almost exclusively uses mature forests and often moves into nearby riparian habitats post‐harvest (Chapin et al. 1998). Riparian specialists, such as American mink (*Neogale vison*), might therefore experience greater competition with marten after forest harvesting (Hodder et al. 2017; Kiseleva 2012).

The habitat characteristics of harvested forests may also influence interspecific interactions (Delheimer et al. 2023; Wiebe et al. 2014). For example, harvested stands often have less complex structure compared with mature forest and may not provide the fine‐scale habitat features required by some species for protective or thermal cover (Barbeito et al. 2009; Seip et al. 2018; Wiebe et al. 2014) or rest sites and dens (Weir et al. 2012). The presence of wildlife species in harvested stands is influenced by the characteristics of trees, such as height, species, density, age, and condition (McComb 2016). Dense, tall stands block sunlight from reaching the ground and reduce shrub cover (West et al. 1981) whereas sparse, open stands facilitate growth of the shrub community and regenerating trees, creating near‐ground cover and browsing opportunities for herbivores (Halls and Alcaniz 1968; West et al. 1981). Downed wood, or coarse woody debris (CWD), can provide cover from predators or access to prey (Wiebe et al. 2014). Dead standing snags provide cavities for dens as well as nests, resting sites, and security cover (Edworthy et al. 2018). These forest attributes contribute to the structural diversity of habitat, both vertically and horizontally, and increase the potential for species to co‐occur.

Mesocarnivores are a diverse guild of species with considerable potential for competitive interspecific interactions due to similar dietary and habitat needs (Roemer et al. 2009). Moreover, mesocarnivores can be sensitive to habitat change, including forest harvesting, and many undergo cyclical population dynamics (Chapin et al. 1998; Linnell et al. 2017; Sullivan and Sullivan 2021). These circumstances provide an opportunity to better understand the effects of population changes and forest harvesting on spatial niche partitioning among species.

We used a long‐term dataset to investigate patterns of co‐occurrence among sympatric mesocarnivores in a harvested forest landscape in central British Columbia, Canada. Our dataset allowed for an analysis of changes in space use during two contrasting periods when the abundance of predators and their prey differed. We used camera traps and Light Detection and Ranging (LiDAR) data to evaluate fine‐scale winter habitat co‐occurrence patterns of four forest‐dwelling mustelids including American marten, American mink, short‐tailed weasel ( *Mustela erminea* ), and fisher ( *Pekania pennanti* ). These sympatric species often have similar diets (Breault et al. 2023) and habitat needs (Evans and Mortelliti 2022; Hodder et al. 2017; Manlick et al. 2017; Suffice et al. 2020) that may increase spatial co‐occurrence and the potential for competitive interactions (Murray et al. 2023; Sanglas and Palomares 2022). We used fine‐scale LiDAR‐derived forest inventory data to facilitate investigations of animal occurrence at spatial scales similar to animal movements. Locally, fishers were rarely detected until 2020, and this change allowed us to study the interactions of other species before and during the increase of this relatively uncommon species in the community.

Our objective was to investigate ecological factors influencing the habitat use and co‐occurrence of four mustelid species (marten, mink, fisher, and weasel) during two contrasting periods in the composition of the prey and predator community. We hypothesized that (1) habitat covariates associated with the occupancy of the four mustelid species would be consistent among years and with previous literature on habitat use of each species. For example, we predicted that the weasel would occupy habitats with greater volumes of CWD (Linnell et al. 2017), mink would occupy habitats closer to riparian areas (Hodder et al. 2017), marten would occupy mature forest with greater canopy closure above 10 m (Lofroth 1993), and fisher would occupy habitats with shallower snow (Krohn et al. 1995). We tested this first hypothesis using single‐species occupancy models. We hypothesized (2) that the habitat covariates associated with co‐occupancy of short‐tailed weasel, mink, and marten would differ in years with high fisher abundance. We predicted that when fisher detections were low (2015–2016), short‐tailed weasels, mink, and marten would use habitats later occupied by fisher and would use these habitats less when fisher detections increased (2020–2022). Finally, because this system is within a disturbed landscape, we hypothesized that (3) the focal species would be more likely to co‐occur in riparian habitat, which is retained during timber harvest. We tested these last two hypotheses using multispecies occupancy models.

## 2 Methods

### 2.1 Study Area

This study took place in and adjacent to the John Prince Research Forest (JPRF), encompassing an area of approximately 350 km^2^ of mixed‐wood stands between 54°35′–54°45′ N latitude and 124°10′–124°36′ W longitude. The portion of the study area adjacent to the JPRF extended beyond the research forest to the north, an area with more intensive forest harvesting. The study area is comprised of several forest types in different seral stages. Most forest stand types are dominated by Douglas fir (*Pseudotsuga menziesii var glauca*), lodgepole pine ( *Pinus contorta* ), hybrid white spruce ( *Picea glauca* × *engelmannii*), subalpine fir ( *Abies lasiocarpa* ), and interspersed with smaller patches of deciduous stands dominated by trembling aspen ( *Populus tremuloides* ), black cottonwood ( *Populus trichocarpa* ), and paper birch ( *Betula papyrifera* ). The area ranged in elevation from 700 to 1267 m above sea level and experienced short, warm summers (average temperature 17°C) and long, cold winters (average temperature −10°C) with an average snowpack between 0.80 and 1.2 m. Riparian features in the study area included large lakes, Tezzeron Lake (7989.4 ha) and Pinchi Lake (5554.2 ha), many small lakes, wetlands, and streams. The study area has a history of logging dating back to the 1940s. Following the establishment of the JPRF in 1999, logging continued at a smaller scale using practices that often led to the retention of features associated with wildlife habitat, such as connectivity among patches, CWD, and wildlife trees. Past and current logging practices created forests with a mosaic of stand ages, tree composition, and structural complexity in the study area.

The JPRF supports a wide range of wildlife species, including a diverse group of mesocarnivores such as wolverine ( *Gulo gulo* ), red fox ( *Vulpes vulpes* ), coyote ( *Canis latrans* ), Canada lynx ( *Lynx canadensis* ), fisher, American marten, American mink, and short‐tailed weasel. All the mesocarnivores are listed as “Least Concern” on the International Union for Conservation of Nature's ranking (IUCN, n.d.). Monitoring data between 2015 and 2022 indicated that these species had experienced shifts in population densities, notably a reduction in abundance of Canada lynx (Crowley et al. n.d.). Lynx declines may be linked to decreased abundance of snowshoe hares (Chisholm 2023; Crowley et al. n.d.; Krebs et al. 2018). Other species, such as marten and mink, also experienced declines in detections during this period, whereas the number of detections of wolverine, fisher, and short‐tailed weasel increased.

### 2.2 Field Data Collection

**FIGURE 1:**
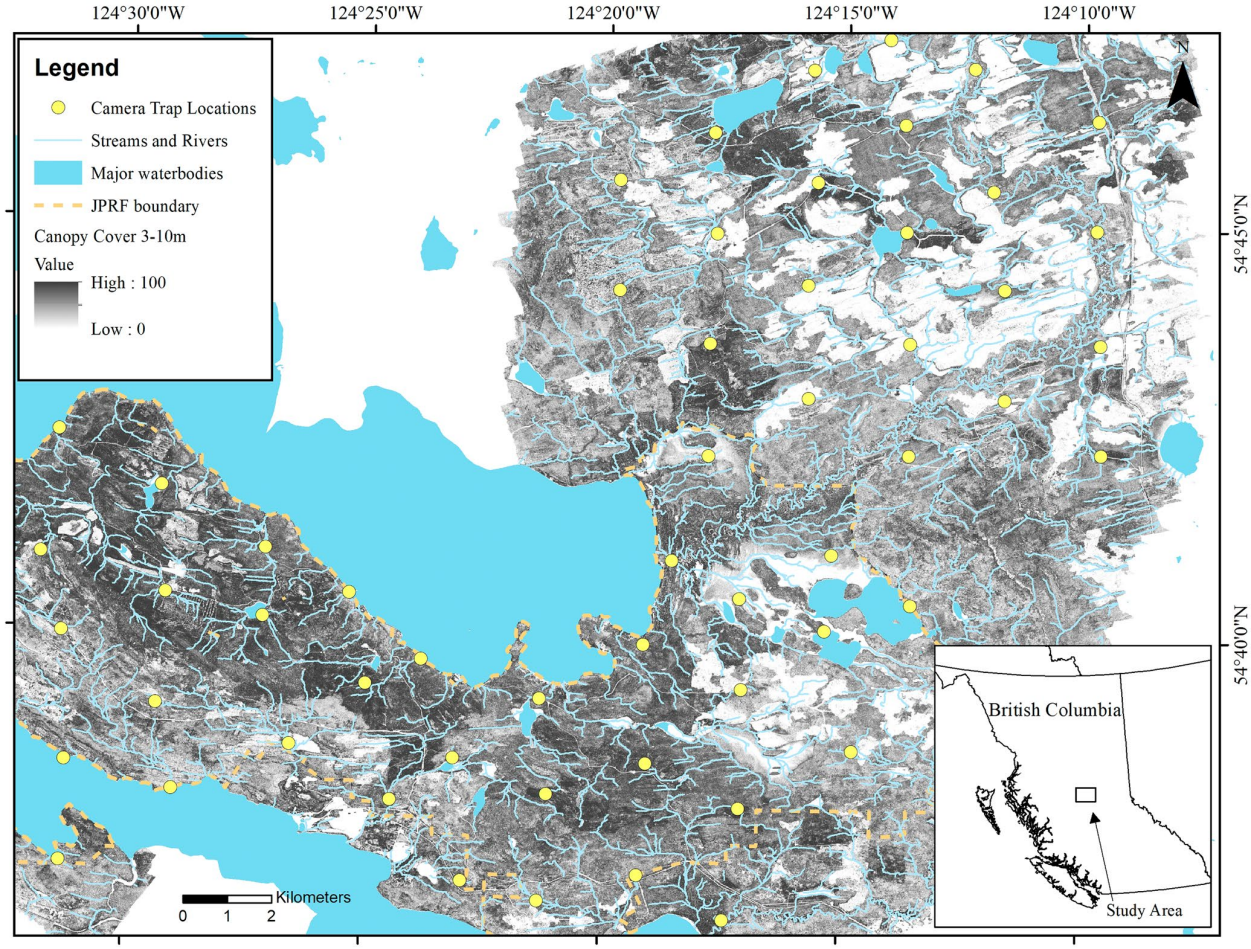
Camera trap locations in the John Prince Research Forest, British Columbia, Canada. Also shown are all major waterbodies, rivers, and streams in blue. More darkly shaded areas depict more canopy cover between 3 and 10 m, while more lightly shaded areas depict less canopy cover between 3 and 10 m.

**FIGURE 2:**
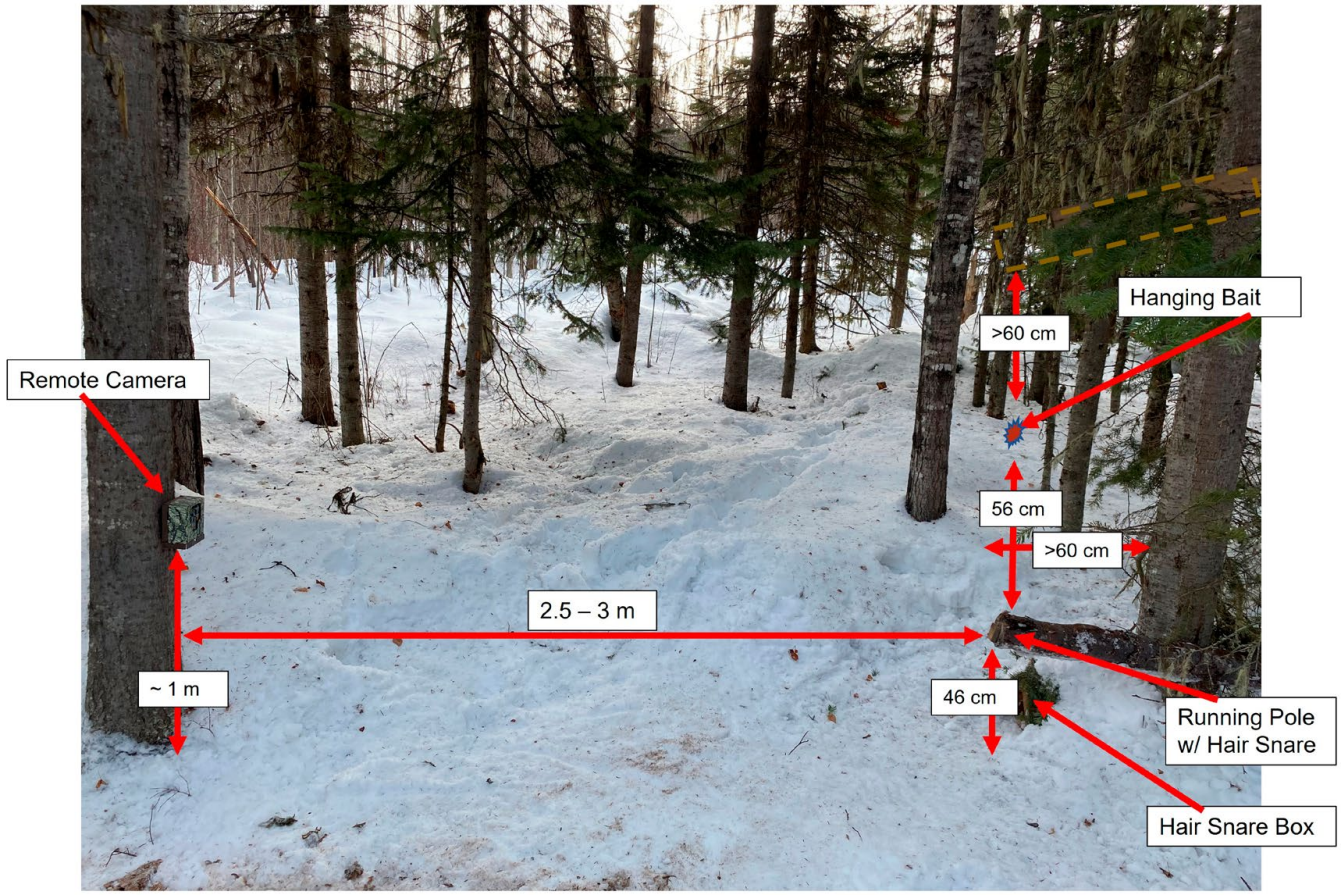
Typical camera trap set‐up, including measurements between camera and bait set, within the John Prince Research Forest, BC, Canada.

The JPRF has operated a camera grid of 66 cameras since 2015 to observe mammal communities (Figure 1). The cameras were active during two separate sampling periods, 2015–2016 and 2020–2022. The camera grid spanned the extent of the research forest, with each hexagonal grid cell covering an area of 5.41 km^2^. Cameras were placed as close as possible to the center of each of the 66 hexagons. In hexagons where the center fell in a lake or other inaccessible area, cameras were placed at the nearest accessible location. Each camera was mounted to a tree approximately 1 m off the ground, angled slightly downward, and 3 m away from a hanging bait, which included a small piece of beaver meat and a scent lure (Figure 2). The cameras (Bushnell Trophy Cam model 119467 and Bushnell Trophy Cam HD Max Model 119477 [2015–2016], Browning Dark Ops HD Pro Trail Cameras Model BTC‐6HDP; Browning, Utah, USA; [2020–2022]) were set to record a video for 10 s when triggered by movement. The cameras were checked and the sites rebaited every 14 days in the winter.

Trained technicians watched every video and recorded the species, the date, and the time observed in the video. For analyses presented here, we used data from February 1 to April 15 of each year. A session was described as the number of days between researcher visits to a camera (winter sessions were 14 days). A camera that was in operation for more than 50% of the days during the 14‐day session was considered “active.” Cameras active for less than 50% of days within a session were excluded from that session, and cameras with less than 50% active sessions were removed from the analyses for that year. Camera effort was then calculated by dividing the total number of cameras deployed by the number of active cameras available for the entire session.

### 2.3 Covariates

The dependent variable in the models was the presence (1) or absence (0) of a species during 2‐week sampling occasions, obtained through empirical data within this study. For the independent variables, we used LiDAR data and field measurements to quantify seven covariates that we hypothesized would characterize the habitat of the focal species to estimate occupancy (Table 1). Temperature and time since bait were included as detection covariates in our occupancy models (Table 1).

**TABLE 1:** Detection and occupancy covariates used for modeling occupancy of short‐tailed weasel ( *Mustela erminea* ), American mink (Neogale vison), American marten ( *Martes americana* ), and fisher ( *Pekania pennanti* ) in the John Prince Research Forest, British Columbia, Canada, during the winters of 2015–2016 and 2020–2022.

	Covariate name	Source	Range
*Detection covariates and units*			
Temperature (C°)	temp	Stuart Lake Weather Station	−15.9 to 7.3
Average time with bait (days)	bait	Camera detections	2.2–17.5
*Occupancy covariates and units*			
Vegetation cover between 0 and 3 m (%)	cc0‐3	LiDAR	18.3–83.2
Vegetation cover between 3 and 10 m (%)	cc3‐10	LiDAR	24.3–92.7
Vegetation cover above 10 m (%)	cc10	LiDAR	0–87.6
Distance to nearest riparian feature (m)	rip_dist	LiDAR	0.01–445.5
Dominant tree type	tree_type	Field measurements	—
Coarse woody debris (m^3^)	cwd	Field measurements	1.8–240.6
Snow depth (cm)	sd	Field measurements	21.0–85.9

We considered temperature and time since bait as detection covariates in our occupancy models. Temperature data were collected from Environment Canada's National Climate Data and Information Archive as the average temperature for the area for each session. Temperature may influence detections in different ways. For example, animals may reduce movements during warm temperatures if the snowpack becomes soft and difficult to move through or during cold temperatures to conserve energy (Zalewski 2001). We used bait and scent lure to increase the likelihood of detections, but if the bait was consumed early in the session, the likelihood of detection would decrease over time. For the bait covariate, we calculated the number of days since a site was baited by calendar day and then averaged these values for each 2‐week session. Since all sites were not baited on the same day of a 2‐week occasion, and the start of occasions used in the occupancy analysis did not always perfectly coincide with camera check days, we used an average number of days since each site was baited as a covariate.

We used seven variables to represent various aspects of habitat. Occupancy covariates were derived from LiDAR or field measurements. LiDAR data were collected between August 14 and September 10, 2015, with an average realized pulse density of 8–10 pulses/m^2^. These data provided a detailed image of the landscape and were refined into metrics that represented vegetation structure at the time of data collection.

Covariates were extracted from a circle with a 50‐m radius centered on the camera location. These covariates included representations of canopy closure for vertical layers in the vegetation ranging in height above ground from 0 to 3 m, 3 to 10 m, and above 10 m, as well as distance to riparian features. The vegetation cover covariates described the complexity of the stand in each height class; a larger value represented more complexity at that height above ground. The vegetation cover measurements at 3–10 m and above 10 m were corrected for winter conditions (without leaves) in deciduous‐dominant sites as LiDAR data were collected during summer (Crowley et al. n.d., *In review*). Vegetation cover in the 0–3 m height class reflected the relative amount of low shrubs and regenerating trees. Vegetation cover in the 3–10 m height class represented tall shrubs and regenerating trees that were more than 3 m in height. Notably, vegetation cover between 3 and 10 m represented multiple habitats, primarily regenerating coniferous plantations and shrubby riparian forests. Vegetation cover above 10 m represented the cover from taller and mature trees. Distance to riparian habitats described how close the camera location was to riparian features including small streams, rivers, wetlands, or lakes.

Covariates reflecting tree composition, CWD, and snow conditions were recorded at each camera site. Tree composition was classified as deciduous, coniferous, or mixed based on dominant trees within a circular plot with an 11.28‐m radius centered on the camera site. Sites were classified as deciduous if > 75% of the trees within the plot were deciduous and coniferous if > 75% of trees within the plot were coniferous. Sites with < 75% of either type of tree were categorized as mixed. CWD volume was measured at each site using three 50‐m long transects radiating out from the camera location (Stevens 1997). A permanent measuring stick was placed in the snow for the duration of the survey, and snow depth was recorded every 2 weeks in 2020, 2021, and 2022. We used tolerance scores to assess covariates for excessive collinearity (Menard 2002) with a threshold of 0.1. Scores for variables in all models were > 0.1, which indicates that no covariates were collinear.

### 2.4 Occupancy Models

#### 2.4.1 Single Species Occupancy Models

We used single‐season, single‐species occupancy models to test our first hypothesis, that habitats associated with occupancy of the four focal species (i.e., weasel, mink, marten, and fisher) would vary among years. We fit models using the R package “unmarked” (Fiske and Chandler 2011). A species was considered present if detected on one or more days during a 2‐week session. Weasel occurred at a large proportion of sites across years (e.g., 61 out of 66 sites in 2022). This high naïve occupancy rate caused several of the models to be overparameterized or non‐convergent. Accordingly, we developed an adapted index of presence or absence to correct for the large number of detections. Specifically, in 2015, 2016, 2020, and 2021, weasels were considered absent at sites with zero or one detections within a 2‐week session and present at sites with two or more detections. In 2022, weasels were considered absent at sites with < 3 detections within a 2‐week session, and present at sites with ≥ 3 detections. This correction still included 45 of 66 (68% naïve occupancy) sites with detections in 2022. This correction better represented the core habitat of weasel by distinguishing between habitats where weasel were found at high versus low densities. Fishers were detected only once in each of 2015 and 2016, so only models for 2020, 2021, and 2022 were built for fisher.

We defined a set of 12 models that represented a priori hypotheses explaining habitat occupancy of the focal species, in addition to a null model (Table 2). These models were selected to best represent distinct forest types found in the study area, characterized by different forest harvesting methods and intensity. Models for each year and species were fit separately using the “unmarked” package in R and then compared to the null model using the Akaike information criterion with a correction for small sample size (AIC_*c*_) (Mazerolle 2023). Before fitting the occupancy covariates, we identified detection covariates by comparing three models with either temperature, time since bait, or a constant on the detection parameter while keeping the occupancy parameter constant. We did not use temperature and time since bait together to avoid model overparameterization. The covariates from the highest ranked AIC_*c*_ model for detection were used with subsequent models that were defined to evaluate different combinations of occupancy covariates. Occupancy covariates from models that held 75% of the model weights were considered to have substantial support and were used in subsequent multispecies modeling.

**TABLE 2:** Single‐species occupancy models for short‐tailed weasel ( *Mustela erminea* ), American mink (*Neogale vison*), American marten ( *Martes americana* ), and fisher ( *Pekania pennanti* ) in 2015, 2016, 2020, 2021, and 2022 in the John Prince Research Forest, British Columbia, Canada.

Model name	Covariates included
Full habitat model	cc0_3 + cc3_10 + cc10 + rip_dist + tree_type + cwd
Vertical structure	cwd + cc0_3 + cc3_10 + cc10
Big tree characteristics	cc10 + tree_type
Riparian + structure	cc0_3 + cc3_10 + rip_dist
Type of riparian	rip_dist + tree_type
Ground‐ to mid‐story structure	cc0_3 + cc3_10
Riparian	rip_dist
Ground cover	cc0_3
Mid‐story cover	cc3_10
Mature stand canopy cover	cc10
Coarse woody debris	cwd
Snow depth	sd
Null	(no occupancy covariates)

We used the Mackenzie‐Bailey goodness of fit test on the single‐species models to determine if the highest ranked models fit the data adequately (Mackenzie and Bailey 2004). If the *c*‐hat value of the global model was above the threshold, the models were ranked using QAIC_*c*_ to address overdispersion of the data. We used 95% confidence intervals to assess the strength of effect of each predictor covariate on the dependent variable.

#### 2.4.2 Multispecies Occupancy Models

To evaluate Hypotheses 2 (species co‐occurrence related to fisher abundance) and 3 (species co‐occurrence in riparian habitat), we used the highest AIC_*c*_‐ranked single‐species occupancy models from Hypothesis 1. We also included distance to riparian and mid‐story cover (cc3–10 m) to test Hypothesis 3 (Table 3). We combined the winter seasons in each of the two ecological periods to investigate differences in co‐occurrence patterns. Conditional two‐species models were built for the winters of 2015 and 2016 and for the winters of 2020, 2021, and 2022. Species detections were combined for the years included in each season (2015/2016 and 2020/2021/2022). These conditional models were first fit to determine if the detection probability of a species was dependent or independent of the other species. Once dependence was determined, models were fit to determine which detection covariates to use in the models. Models were compared by fitting three detection models (null, time since bait, and temperature), all while keeping the occupancy covariate constant. These detection models were ranked based on AICc scores and were used in consequent occupancy models. The multispecies occupancy models produced three types of results for each model, and each scenario was presented for each of the covariates within the model. Scenario 3 was used to define species co‐occurrence. Covariates were considered significant if *p* ≤ 0.05. We used 95% confidence intervals to assess the strength of effect of each predictor covariate on the dependent variable.

- Scenario 1: The probability of occupancy of species one where species two was absent.
- Scenario 2: The probability of occupancy of species two where species one was absent.
- Scenario 3: The probability that the area is occupied by both species.

**TABLE 3:** Multispecies occupancy models for each survey period (2015–2016 and 2020–2022) and species pairings between short‐tailed weasel ( *Mustela erminea* ), American mink (Neogale vison), American marten ( *Martes americana* ), and fisher ( *Pekania pennanti* ) in the John Prince Research Forest, British Columbia, Canada.

Species pair	Years	Top single‐species models included in each multispecies framework
Marten and Weasel	2015–2016	Mid‐story cover, ground‐ to mid‐story cover, mature stand canopy cover, riparian
Marten and Mink	2015–2016	Mid‐story cover, ground cover, mature stand canopy cover, riparian
Mink and Weasel	2015–2016	Mid‐story cover, ground cover, riparian
Fisher and Mink	2020–2022	Mid‐story cover, riparian, snow depth
Fisher and Marten	2020–2022	Mid‐story cover, ground cover, mature stand canopy cover, snow depth
Fisher and Weasel	2020–2022	Mid‐story cover, riparian, mature stand canopy cover, snow depth
Marten and Weasel	2020–2022	Mid‐story cover, ground cover, riparian
Marten and Mink	2020–2022	Mid‐story cover, riparian
Mink and Weasel	2020–2022	Mid‐story cover, mature stand canopy cover, riparian

We ranked models based on AICc scores for small sample sizes (Fiske and Chandler 2011; Mazerolle 2023) and compared all multispecies models to the null model.

## 3 Results

From the 66 cameras, 13,971 videos were captured of the four focal species between February 1 and April 15 of 2015, 2016, 2020, 2021, and 2022. The average camera effort was 94% active across all years, with 2% of the sessions removed from the analysis in 2015, 2016, 2020, 2021, and 7% in 2022. In 2022, three camera sites were active for less than 50% of sessions; no data from those camera sites were included in the analysis. There were 269 videos of fisher, resulting in 64 independent daily detections at 41 sites (Table 4). Two fisher detections, one in 2015 and another in 2016, were not used in the analyses. There were 5188 videos of marten, resulting in 377 independent daily detections at 55 sites, and 432 videos of mink, resulting in 76 independent daily detections at 30 sites (Table 4). There were 8082 videos of weasel, resulting in 349 independent daily detections at all 66 sites. Once weasel detections were re‐classified into high versus low occurrence, there were 242 independent daily detections at 41 sites (Table 4).

**TABLE 4:** Number of independent daily detections and unique sites where fisher ( *Pekania pennanti* ), American marten ( *Martes americana* ), American mink (*Neogale vison*), and short‐tailed weasel ( *Mustela erminea* ) were detected in the John Prince Research Forest, British Columbia, Canada during a snowshoe hare ( *Lepus americanus* ) population high (2015–2016) and low (2020–2022).

Species	2015–2016		2020–2022	
	Independent daily detections	Number of sites	Independent daily detections	Number of sites
Fisher	2	2	64	41
Marten	200	41	177	47
Mink	39	28	37	20
Weasel	106	63	243	66
Weasel (reclassified)	48	31	194	41

### 3.1 Detection Covariates Identified Using Single‐Species Occupancy Models

Of 18 species‐year combinations, the null model for detection was ranked highest 10 times. The model with temperature as a detection covariate was ranked highest seven times, four times for weasel, once for mink, and twice for marten. The direction of the effect of temperature varied among years and species; weasels were more likely to be detected when temperatures were warmer in 2020, 2021, and 2022 and less likely to be detected when temperatures were warmer in 2015. Mink were less likely to be detected when temperatures were warmer in 2016. Marten were more likely to be detected when temperatures were warmer in 2020 and 2021. Time since bait was identified as a detection covariate in only one model—for weasel detections in 2016. Weasels were less likely to be detected the longer the time since bait was added to the site; however, the effect was not significant.

### 3.2 Habitat Covariates Associated With Occupancy Among Years for Each Species

We used single‐species occupancy models to test hypothesis 1, which was that habitat covariates associated with occupancy would be similar among years and reflect species‐specific habitat requirements. Overall, models that included mid‐canopy vegetation cover (3–10 m) and distance to riparian tended to rank highest across species and years; however, the associations were generally weak and varied among years and species (Table 4). Complete model sets and their AIC rankings for each species and each year are listed in Table S1.

The null model for occupancy ranked highest for weasels in 4 of the 5 years and was within the top model set (ΔAIC*c* > 2) in all years, indicating that no covariates explained more variation in occupancy than expected due to chance alone. In 2016, the “mid‐story cover” model ranked highest. Specifically, weasel prevalence was positively associated with sites that had greater amounts of canopy cover between 3 and 10 m; however, the association was not significant (Table 4).

The “riparian” model was ranked highest for mink in four of the 5 years of the study. Overall, mink were less likely to occupy sites at greater distances from riparian, although the relationship was not statistically significant in 2015 and 2021 (Table 4). In 2016, the “mid‐story cover” model was ranked highest. Mink were less likely to occupy sites with greater mid‐canopy complexity (vegetation cover between 3 and 10 m), but the relationship was not significant. The null model ranked outside the top model set (ΔAIC*c* > 2) in all years except in 2020, indicating that the top models explained more variation in co‐occupancy than expected due to chance alone in those years.

Marten were less likely to occupy sites with greater vegetation cover between 3 and 10 m in 2 of 5 years; coefficients for this relationship were significant in all years, and the null model ranked below the top model set (ΔAIC*c* > 2) in 2016, 2021, and 2022. Marten were also less likely to occupy sites with greater proportions of vegetation cover between 0 and 3 m in 1 of 5 years, but the relationship was only significant in 2015.

The top models for fisher were “snow depth” in 2020, “riparian” in 2021, and “snow depth and riparian” in 2022 (Table 4). Overall, fishers were less likely to occupy sites farther from riparian and with greater snow depths; the only significant coefficient, however, was distance to riparian in 2021. The null model ranked in the top model set (ΔAICc < 2) in 2020 and 2022.

### 3.3 Habitat Variables Associated With Species Co‐Occurrence During Periods of High and Low Fisher Abundance

Multispecies occupancy models were used to test the second hypothesis, which was that habitat covariates that influenced co‐occurrence of mink, marten, and weasel would differ during two time periods with contrasting fisher abundance. Overall, similar habitat covariates were associated with species co‐occurrence in 2015–2016 and in 2020–2022. Moreover, the direction of the relationships with the covariates (positive or negative) was also relatively consistent between periods. Complete model sets and rankings for each model for each species pair and time period are listed in Table S2.

Models with vegetation cover between 3 and 10 m ranked highest for the co‐occurrence of marten and weasel in both time periods. The highest‐ranked model was the only model in the top model set and had an AIC*c* score > 13 units below the null model in both time periods (Table 5). Marten and weasel were more likely to co‐occur at sites with less vegetation cover between 3 and 10 m, but this relationship was not significant in either period.

**TABLE 5:** Coefficients and 95% confidence intervals (in brackets) for detection (ρ) and occupancy (ψ) parameters, AIC_c_ ranking, and model weight from single species, single season occupancy models built for short‐tailed weasel ( *Mustela erminea* ), American mink (*Neogale vison*), American marten ( *Martes americana* ), and fisher ( *Pekania pennanti* ) using detections from cameras deployed in the winters of 2015, 2016, 2020, 2021, and 2022 in the John Prince Research Forest, British Columbia, Canada.

Species	Year	Model	ρ(temp)	ρ(bait_time)	ψ(cc0–3)	ψ(cc3‐10)	ψ(cc10)	ψ(rip_dist)	ψ(cwd)	ψ(sd)	AIC_*c*_	ω_*i*_
Weasel	2015	ρ (temp) ψ (.)	−0.16 (−0.002, 0.18)								57.33*	0.31
	2016	ρ (bait_time) ψ (cc3‐10)		−0.16 (−0.28, −0.04)		0.03 (−0.007, 0.07)					163.4*	0.29
	2020	ρ (temp) ψ (.)	0.53 (0.11, 0.95)								189.76	0.23
	2021	ρ (temp) ψ (.)	0.13 (0.05, 0.2)								169.27*	0.21
	2022	ρ (temp) ψ (.)	**0.09** (−0.003, 0.18)								162.82*	0.26
Mink	2015	ρ (.) ψ (rip_dist)						−0.04 (−0.07, −0.50)			149.51	0.55
	2016	ρ (temp) ψ (cc3‐10)	**−0.27** (−0.51, −0.03)			−0.1 (−0.24, 0.04)					114.38	0.42
	2020	ρ (.) ψ (rip_dist)						**−0.04** (−0.11, 0.03)			86.75	0.2
	2021	ρ (.) ψ (rip_dist)						−0.1 (−0.26, 0.06)			41.24	0.57
	2022	ρ (.) ψ (rip_dist)						**−0.07** (−0.12, −0.01)			138.11	0.31
Marten	2015	ρ (.) ψ (cc0‐3)			**−0.07** (−0.12, −0.02)						84.53*	0.22
	2016	ρ (.) ψ (cc3‐10)				**−0.09** (−0.15, −0.02)					166.68*	0.50
	2020	ρ (temp) ψ (cwd)	**0.37** (0.05, 0.69)						0.01 (−0.002, 0.03)		174.30*	0.20
	2021	ρ (temp) ψ (rip_dist)	**0.38** (0.03, 0.73)					**−0.05** (−0.09, −0.003)			91.56*	0.42
	2022	ρ (.) ψ (cc3‐10)				**−0.05** (−0.09, −0.01)					166.39*	0.21
Fisher	2020	ρ (.) ψ (sd)								−0.04 (−0.1, 0.02)	166.57	0.25
	2021	ρ (.) ψ (rip_dist)						**−0.05** (−0.09, −0.003)			128.3	0.4
	2022	ρ (.) ψ (rip_dist +SD)						−0.04 (−0.09, 0.02)		−0.16 (−0.39, 0.07)	154.22	0.26

*Note:* Models shown were the highest AIC_c_ ranked of candidate models compared for each species and year. Models with a (.) are the null models. Coefficients in bold font were statistically significant (*p* < 0.05). AIC_c_ values with a (*) are representative of QAIC_c_ values.

The highest‐ranked model for mink and marten in both 2015–2016 and 2020–2022 was vegetation cover between 3 and 10 m (Table 5). Sites where both mink and marten were detected had greater vegetation cover between 3 and 10 m. In both periods, sites with marten but not mink had a negative association with vegetation cover between 3 and 10 m (2015–2016 *p* = 0.09, 2020–2022 *p* = 0.004). The null model in both time periods had ΔAIC*c* greater than 2.

The highest‐ranked model for mink and weasel included vegetation cover between 3 and 10 m in 2015–2016 and distance to riparian in 2020–2022 (Table 5). In 2015–2016, mink and weasel were more likely to co‐occur at sites with lower vegetation cover between 3 and 10 m (*p* = 0.05), whereas in 2020–2022, mink and weasel were more likely to co‐occur at sites farther from riparian habitats. The ΔAIC*c* score for the null model was greater than 2 in both years.

### 3.4 Riparian Co‐Occurrence

We used the same two‐species occupancy models as described above to evaluate our third hypothesis, which was that species pairs would be more likely to co‐occur in riparian habitat. Distance to riparian was ranked among the top models (ΔAIC*c* < 2) for three of nine model sets comparing species pairs, all in 2020–2022 (Table 6). Although none of the associations between co‐occurrence and distance to riparian were significant, the direction of the relationship supported our hypothesis in two of the three cases. Specifically, fisher and mink were more likely to co‐occur at sites closer to riparian habitats (Table 6). Similarly, mink and marten were more likely to co‐occur at sites closer to riparian. By contrast, mink and weasel were more likely to co‐occur at sites farther from riparian (Table 6).

**TABLE 6:** Coefficients and 95% confidence intervals (in brackets) for detection (ρ) and occupancy (ψ) parameters, AIC_c_ ranking, and model weight from multispecies, multi‐season occupancy models built for short‐tailed weasel ( *Mustela erminea* ) (W), American mink (*Neogale vison*) (MK), American marten ( *Martes americana* ) (MN), and fisher ( *Pekania pennanti* ) (FR) using detections from cameras deployed in the winters of 2015, 2016, 2020, 2021, and 2022 in the John Prince Research Forest, British Columbia, Canada.

Species	Year	Formula	Model output	cc0‐3	cc3‐10	cc10	Riparian	AIC*c*	ΔAIC*c*	AIC*c* ω_*i*_
MN + W	2015–2016	ρ(.)ρ(.)ψ(cc3_10 + cc0_3)	MN + W	**0.2** (−0.003, 0.46)	−0.07 (−0.21, 0.06)			850.59	0	0.89
			MN no W	**−0.3** (−0.57, −0.09)	−0.05 (−0.15, 0.03)					
			W no MN	−0.2 (−0.42, 0.03)	0.08 (−0.04, 0.21)					
	2020–2022	ρ(.) ρ(.) ψ (cc3_10)	MN + W		−0.03 (−0.13, 0.06)			1771.12	0	1
			MN no W		−0.04 (−0.12, 0.04)					
			W no MN		−0.02 (−0.10, 0.06)					
MN + MK	2015–2016	ρ(.)ρ(.) ψ cc3_10	MN + MK		0.1 (−0.11, 0.32)			819.11	0	0.81
			MN no MK		−0.1 (−0.33, 0.03)					
			MK no MN		−0.1 (−0.36, 0.10)					
	2020–2022	ρ(.) ρ(.) ψ (cc3_10)	MN + MK		0.08 (−0.02, 0.18)			1164.73	0	0.5
			MN no MK		**−0.09** (−0.15, −0.03)					
			MK no MN		−0.07 (−0.15, 0.01)					
		ρ(.) ρ(.) ψ (dist_rip)	MN + MK				−0.05 (−0.10, 0.010)	1165.03	0.29	0.43
			MN no MK				0.009 (−0.007, 0.03)			
			MK no MN				−0.005 (−0.04, 0.03)			
MK + W	2015–2016	ρ(.) ρ(.) ψ cc3_10	MK + W		**−0.1** (−0.29, 0.002)			594.82	0	0.63
			MK no W		0.04 (−0.045, 0.13)					
			W no MK		0.1 (−0.005, 0.21)					
	2020–2022	ρ(.) ρ(.) ψ (dist_rip)	MK + W				0.2 (−0.23, 0.71)	1173.89	0	0.99
			MK no W				−0.3 (−0.74, 0.20)			
			W no MK				0.007 (−0.009, 0.02)			
FR + MK	2020–2022	ρ(.) ρ(.) ψ (dist_rip)	FR + MK				−0.2 (−0.37, 0.01)	717.68	0	0.99
			FR no MK				−0.003 (−0.02, 0.01)			
			MK no FR				−0.01 (−0.04, 0.01)			
FR + MN	2020–2022	ρ(.) ρ(.) ψ (cc3_10)	FR + MN		0.04 (−0.08, 0.160)			1316.76	0	0.94
			FR no MN		−0.002 (−0.09, 0.09)					
			MN no FR		**−0.09** (−0.18, −0.01)					
FR + W	2020–2022	ρ(.) ρ(.) ψ (cc10)	FR + W			−0.6 (−139.42, 138.15)		1351.93	0	0.99
			FR no W			0.6 (−139.42, 138.15)				
			W no FR			0.6 (−138.08, 139.35)				

*Note:* Models shown represented 75% of the cumulative model weight of candidate models compared for each species pairing and year. Coefficients in bold font were statistically significant (*p* < 0.05). Covariate abbreviations cc0‐3 represent canopy closure from 0 to 3 m, cc3‐10 represent canopy closure between 3 and 10 m, cc10 represent canopy closure above 10 m, and riparian represent the distance from the camera location to a riparian feature.

## 4 Discussion

This research examined co‐occurrence patterns among mesocarnivores in central British Columbia to gain a deeper understanding of habitat features that facilitate their coexistence. We hypothesized that weasels, marten, mink, and fisher would occupy similar habitats annually while exhibiting spatial niche partitioning. Spatial partitioning was assessed using single‐species occupancy models. The increase in fisher detections did not appear to affect the co‐occurrence of weasel, mink, or marten, which was unexpected. We also found that the co‐occurrence of mustelids was associated with forest structural complexity and/or riparian areas in every year of the study, emphasizing the importance of riparian habitats for these animals.

We predicted that each species would be found in specific habitats over time, as reflected by similar habitat variables being associated with occupied sites in each year. We found that some species occupied sites with similar characteristics over the 5 years, while others were more variable. We saw some inter‐annual variation in the characteristics of the sites that were occupied; however, forest cover and proximity to riparian habitats were the most common habitat covariates explaining single‐species occupancy.

Mink showed the most consistent occupancy patterns among years and were more likely to occupy habitats near riparian sites in 4 of the 5 years of the study. The importance of riparian habitat to mink is consistent with other studies of mink in North America (Ben‐David et al. 1996; Hodder et al. 2018; Schooley et al. 2012). Despite associations with riparian in most years, mink occupancy was not statistically associated with riparian habitat in 2016. Mink might use habitats outside riparian areas when intraspecific competition is high; however, our data do not support this possibility because mink detections did not peak in 2016. Instead, mink might use alternative sites when interspecific competition is high. Notably, lynx (Crowley et al. n.d.) and marten had the greatest number of detections in 2016 and may have influenced the distribution of mink through interference competition. In our study area, mink are dietary generalists and may forage at sites outside riparian areas when competition with marten is elevated (Breault et al. 2023).

In general, marten were less likely to occupy sites with greater mid‐story vegetation cover (i.e., 3–10 m), although the association was only significant in 2016 and 2020. One reason for variation in the importance of mid‐story cover among years is that this variable may describe multiple habitat types, the two most common being regenerating stands and riparian forests. The negative association between marten and regenerating forests was identified through comparisons with the results of the riparian covariates and mapping of the vegetation cover between 3 and 10 m covariate. Marten were more likely to occupy sites closer to riparian features, but less likely to occupy sites with high vegetation cover between 3 and 10 m. We hypothesized that marten would occupy sites with greater vegetation cover above 10 m in every year, reflecting the reported relationship between marten and mature forests (Buskirk 1994). However, we found marten were more likely to avoid young forests than they were to occupy forests with mature characteristics, which is consistent with other studies in North America showing that marten avoid regenerating clear cuts (Fuller and Harrison 2005; Poole et al. 2004). Our findings suggest that marten occupy other habitats, such as areas with mature forest characteristics, to avoid occupying regenerating clear cuts.

Although only significant in 2021, habitat variables in the top‐ranked models of fisher occupancy were consistent with our expectations from the literature in all 3 years when data on fishers were available. Specifically, fishers were less likely to occupy sites with greater snow depths in two of the 3 years, which is consistent with the findings of other studies (Evans and Mortelliti 2022; Krohn et al. 1995). Fisher have difficulty moving and hunting above snow (Powell et al. 2003) and likely forage less efficiently in subnivean environments compared with marten and weasel (Fitzgerald 1977; Jung et al. 2021). Fishers were more likely to be detected at sites closer to riparian areas in two of the 3 years. Female fisher use large diameter trees for maternal dens in the spring (Weir et al. 2012) that are often found in riparian habitats (Heemskerk et al. 2009). Riparian forests share other characteristics with mature forests, including CWD, structurally complex understory, and canopy closure (Weir 1995).

The top‐ranked weasel models were not well differentiated from the null model in any year. The broad distribution and high naïve occupancy rate of weasel may have contributed to the variation in covariates and poor model fit. Surprisingly, weasel did not occupy sites associated with greater volumes of CWD. One explanation may be that weasel were less likely to be detected on camera because there was more cover at sites with greater volumes of CWD. Alternatively, weasel may be habitat generalists, and habitat occupancy may be driven more by prey availability than vegetation characteristics. Moreover, our finding that weasel were more likely to occur in higher prevalence at sites farther away from riparian areas in two of the 5 years contrasted with studies of habitat use by weasel in more arid sites in Oregon (Linnell et al. 2017); New Mexico (Frey and Calkins 2014); and Poland (Zub et al. 2008). One explanation for these differences could be the availability of cover in non‐riparian habitats in our study area. Alternatively, the high diversity of predators and competitors that use riparian habitats in our system could displace weasel. Previous studies have also observed that sexual dimorphism and age affect habitat selection by weasel (Linnell et al. 2017), which also could have influenced our findings, as males and females may use different habitats, adding noise to these results.

We hypothesized that an increase in the fisher population from 2015 to 2022 would cause a top‐down trophic cascade in our system (Wallach et al. 2015), either directly through predation or indirectly through competition for shared resources. Yet, the increase in fisher detections did not appear to affect the co‐occurrence patterns among marten, mink, and weasel. Fisher occasionally eat marten, short‐tailed weasel, and other fishers (Weir et al. 2005), and has extensive dietary overlap with marten (Manlick et al. 2017). Dietary competition may occur between fisher and other species in our study area, but there is no description of the diet of fishers in our system. Spatial analysis alone cannot completely quantify competition in these complex systems (Murray et al. 2023). Although patterns of co‐occurrence did not change when the fisher increased, the temporal activity patterns of species may have changed to avoid negative interactions with fisher (Kupferman et al. 2021). Further studies of co‐occurrence relationships should include analyses of temporal activity patterns.

Between 2015–2016 and 2020–2022, the number of detections of marten and mink decreased, but detections of weasel increased. The number of detections can be used as a rough estimate of abundance (Kenney et al. 2024; Mace et al. 1994), but further study is required to determine if the increase in fisher led to a decrease in marten and mink, and a possible release of weasel. In addition, this system experienced a steep decline in both snowshoe hares ( *Lepus americanus* ) and Canada lynx ( *Lynx canadensis* ) between 2015 and 2022 (Chisholm 2023; Crowley et al. n.d.). Cyclical population changes, synchronous with the snowshoe hare cycle, have been observed for mink and weasel in Alberta (Keith and Cary 1991) and other species in the Yukon (Boutin et al. 1995). This large shift in both the predator and prey community may have contributed to the changes in mustelid detections in this study.

Our results show that mink, marten, and fisher co‐occurred closer to riparian features, which aligns with our third hypothesis. By contrast, weasel had lower detection rates at sites with mink, marten, and fisher in riparian areas. Moreover, weasel occurred at lower detection rates at sites closer to riparian habitat even in the absence of competitors. Possible reasons for fewer detections of weasel in riparian areas include increased risk of predation by fisher (Weir et al. 2005), competition for prey with marten (Breault et al. 2023), or competition for cover with mink. Our findings contrast with those from more arid systems where weasels are often found in riparian habitats in part due to the availability of cover (Doyle 1990; Frey and Calkins 2014; Sullivan and Sullivan 2021). In the temperate system where our study was conducted, there is more cover available outside of riparian areas, which could explain the differences in results among studies. Our results are consistent with other studies that found weasel in BC often use open areas with low structural complexity, such as early‐seral habitat (Mowat et al. 2000).

The co‐occurrence of mink, marten, and fisher at sites closer to riparian features is surprising due to the high dietary overlap among these species (Breault et al. 2023; Hodder et al. 2017; Manlick et al. 2017). Spatial overlap among these species could be facilitated by the complexity of riparian habitat, which may provide opportunities for fine‐scale niche partitioning. Alternatively, these species may avoid direct competition for prey or space if they use riparian areas primarily for travel. Riparian habitats include the land surrounding features such as streams, rivers, and lakeshores, which can serve as travel corridors in winter (Perault and Lomolino 2000; Santos et al. 2011). Regardless of the mechanism, our findings highlight the importance of riparian areas for the co‐occurrence of these three species.

## 5 Conclusion

We found that the habitat occupancy patterns of weasel, mink, marten, and fisher varied among years, but that riparian and forest structure consistently influenced occupancy. Moreover, the vertical position of cover in the canopy also varied in importance and directional association (positive or negative) by species and year. These findings emphasize the importance of managing fine‐scale features of forests, specifically the structural complexity of vertical cover, in promoting occupancy and co‐occurrence of mustelids. In our system, the apparent increase in fisher abundance did not change co‐occurrence patterns among weasel, mink, and marten, suggesting that fisher occurrence did not influence space use by mink and marten. However, we found that the presence of fisher, mink, and marten could affect the use of riparian areas by weasels. Moreover, our occupancy models did not identify habitat variables consistently associated with weasel occupancy, which highlights a need for further assessment of weasel habitat requirements. Overall, our results demonstrate the importance of retaining riparian areas with complex mid‐story vegetation to facilitate the occupancy and co‐occurrence of mustelid species.

## Author Contributions

**Lauren Wheelhouse:** conceptualization (equal), data curation (equal), formal analysis (equal), investigation (equal), methodology (equal), writing – original draft (lead), writing – review and editing (lead). **Heather Bryan:** conceptualization (equal), formal analysis (supporting), supervision (equal), writing – review and editing (equal). **Shannon Crowley:** conceptualization (supporting), data curation (equal), formal analysis (supporting), funding acquisition (equal), writing – review and editing (supporting). **Chris Johnson:** conceptualization (supporting), formal analysis (supporting), methodology (supporting), writing – review and editing (supporting). **Dexter Hodder:** conceptualization (equal), data curation (equal), formal analysis (supporting), project administration (equal), supervision (equal), writing – review and editing (supporting).

## Conflicts of Interest

The authors declare no conflicts of interest.

## Supporting information


**Table S1:** Single‐species, single season occupancy models ranked by AIC*c* (or QAIC*c*, indicated by a *) for fisher ( *Pekania pennanti* ), American marten ( *Martes americana* ), American mink (*Neogale vison*), and short‐tailed weasel ( *Mustela erminea* ) in 2015, 2016, 2020, 2021, and 2022 in the John Prince Research Forest, British Columbia, Canada. Twelve candidate models were run for each species in 2015 and 2016, and fifteen candidate models were run for each species in 2020, 2021, and 2022. Non‐convergent models are not included in this table.

**Table S2:** Multi‐species occupancy models ranked by AIC*c* for fisher ( *Pekania pennanti* ), American marten ( *Martes americana* ), American mink (*Neogale vison*), and short‐tailed weasel ( *Mustela erminea* ) in 2015–2016 and 2020–2022 in the John Prince Research Forest, British Columbia, Canada. Fit 1 describes an intercept only model, assuming independence between species, and fit 2 describes an intercept‐only model that assumes dependence between species. Both fit 1 and fit 2 are considered null models for occupancy.

## Data Availability

The data that support the findings of this study are openly available in the University of Northern British Columbia Dataverse at https://borealisdata.ca/privateurl.xhtml?token=025575bd‐084f‐49fb‐b3ff‐f0bfeb23c6e2.
