# Supplementary material for: Riparian Areas and Fine‐Scale Forest Cover and Structure Drive Occupancy Patterns of Sympatric Mustelids
Source: Ecol Evol. 2025 May 7;15(5):e71370. doi: 10.1002/ece3.71370 (PMC12058454; doi:10.1002/ece3.71370)
Supplement: Supplementary file 1 — Table S1: Single‐species, single season occupancy models ranked by AICc (or QAICc, indicated by a *) for fisher ( Pekania pennanti ), American marten ( Martes americana ), American mink (Neogale vison), and short‐tailed weasel ( Mustela erminea ) in 2015, 2016, 2020, 2021, and 2022 in the John Prince Research Forest, British Columbia, Canada. Twelve candidate models were run for each species in 2015 and 2016, and fifteen candidate models were run for each species in 2020, 2021, and 2022. Non‐convergent models are not included in this table. [file ECE3-15-e71370-s002.docx]

# **Table S1 –** Single-species, single season occupancy models ranked by AIC*c* (or QAIC*c*, indicated by a *) for fisher (*Pekania pennanti*), American marten (*Martes americana*), American mink (*Neogale vison*), and short-tailed weasel (*Mustela erminea*) in 2015, 2016, 2020, 2021, and 2022 in the John Prince Research Forest, British Columbia, Canada. Twelve candidate models were run for each species in 2015 and 2016, and fifteen candidate models were run for each species in 2020, 2021, and 2022. Non-convergent models are not included in this table.

| **Species** | **Year** | **Model Name** | **rank** | **AIC*c*** | Δ**AIC*c*** | **AIC*c*** ω*_i_* |
| --- | --- | --- | --- | --- | --- | --- |
| Weasel | 2015 | Null | 1 | 57.33* | 0 | 0.31 |
| Weasel | 2015 | Ground canopy | 2 | 58.51* | 1.18 | 0.17 |
| Weasel | 2015 | Mature stand canopy cover | 3 | 59.04* | 1.71 | 0.13 |
| Weasel | 2015 | Riparian | 4 | 59.19* | 1.86 | 0.12 |
| Weasel | 2015 | Coarse woody debris | 5 | 59.67* | 2.34 | 0.1 |
| Weasel | 2015 | Ground- to mid- story structure | 6 | 60.39* | 3.06 | 0.07 |
| Weasel | 2015 | Mid-story canopy | 7 | 60.9* | 3.57 | 0.05 |
| Weasel | 2015 | Riparian + structure | 8 | 63.1* | 5.77 | 0.02 |
| Weasel | 2015 | Big tree characteristics | 9 | 63.15* | 5.82 | 0.02 |
| Weasel | 2015 | Type of riparian | 10 | 63.31* | 5.98 | 0.02 |
| Weasel | 2015 | Vertical structure | 11 | 65.09* | 7.76 | 0.01 |
| Weasel | 2016 | Mid-story canopy | 1 | 163.4* | 0 | 0.29 |
| Weasel | 2016 | Null | 2 | 163.56* | 0.15 | 0.27 |
| Weasel | 2016 | Ground- to mid- story structure | 3 | 165.65* | 2.24 | 0.1 |
| Weasel | 2016 | Coarse woody debris | 4 | 165.83* | 2.42 | 0.09 |
| Weasel | 2016 | Ground canopy | 5 | 165.85* | 2.45 | 0.09 |
| Weasel | 2016 | Riparian | 6 | 165.88* | 2.48 | 0.08 |
| Weasel | 2016 | Riparian + structure | 7 | 167.98* | 4.58 | 0.03 |
| Weasel | 2016 | Mature stand canopy cover | 8 | 167.98* | 4.58 | 0.03 |
| Weasel | 2016 | Vertical structure | 9 | 170.34* | 6.94 | 0.01 |
| Weasel | 2016 | Big tree characteristics | 10 | 170.48* | 7.08 | 0.01 |
| Weasel | 2016 | Type of riparian | 11 | 170.63* | 7.23 | 0.01 |
| Weasel | 2016 | Full habitat model | 12 | 177.08* | 13.68 | 0 |
| Weasel | 2020 | Null | 1 | 189.76 | 0 | 0.23 |
| Weasel | 2020 | Ground canopy | 2 | 190.75 | 0.99 | 0.14 |
| Weasel | 2020 | Snow depth | 3 | 191.21 | 1.46 | 0.11 |
| Weasel | 2020 | Coarse woody debris | 4 | 191.23 | 1.47 | 0.11 |
| Weasel | 2020 | Riparian | 5 | 191.57 | 1.81 | 0.09 |
| Weasel | 2020 | Mid-story canopy | 6 | 191.68 | 1.92 | 0.09 |
| Weasel | 2020 | Snow depth and distance to riparian | 7 | 192.75 | 2.99 | 0.05 |
| Weasel | 2020 | Ground- to mid- story structure | 8 | 192.86 | 3.1 | 0.05 |
| Weasel | 2020 | Snow depth and coarse woody debris | 9 | 193.04 | 3.28 | 0.04 |
| Weasel | 2020 | Mature stand canopy cover | 10 | 193.46 | 3.71 | 0.04 |
| Weasel | 2020 | Riparian + structure | 11 | 195.07 | 5.32 | 0.02 |
| Weasel | 2020 | Type of riparian | 12 | 195.69 | 5.93 | 0.01 |
| Weasel | 2020 | Big tree characteristics | 13 | 195.88 | 6.12 | 0.01 |
| Weasel | 2020 | Vertical structure | 14 | 197.12 | 7.36 | 0.01 |
| Weasel | 2020 | Full habitat model | 15 | 203.87 | 14.11 | 0 |
| Weasel | 2021 | Null | 1 | 169.27* | 0 | 0.21 |
| Weasel | 2021 | Riparian | 2 | 169.56* | 0.29 | 0.18 |
| Weasel | 2021 | Ground canopy | 3 | 170.61* | 1.34 | 0.11 |
| Weasel | 2021 | Mid-story canopy | 4 | 171.01* | 1.74 | 0.09 |
| Weasel | 2021 | Snow depth | 5 | 171.36* | 2.09 | 0.07 |
| Weasel | 2021 | Coarse woody debris | 6 | 171.61* | 2.34 | 0.07 |
| Weasel | 2021 | Snow depth and distance to riparian | 7 | 171.74* | 2.48 | 0.06 |
| Weasel | 2021 | Type of riparian | 8 | 171.79* | 2.52 | 0.06 |
| Weasel | 2021 | Ground- to mid- story structure | 9 | 172.61* | 3.34 | 0.04 |
| Weasel | 2021 | Mature stand canopy cover | 10 | 173.4* | 4.13 | 0.03 |
| Weasel | 2021 | Riparian + structure | 11 | 173.56* | 4.29 | 0.02 |
| Weasel | 2021 | Big tree characteristics | 12 | 173.61* | 4.34 | 0.02 |
| Weasel | 2021 | Snow depth and coarse woody debris | 13 | 173.78* | 4.51 | 0.02 |
| Weasel | 2021 | Vertical structure | 14 | 176.98* | 7.71 | 0 |
| Weasel | 2021 | Full habitat model | 15 | 179.55* | 10.28 | 0 |
| Weasel | 2022 | Null | 1 | 162.82* | 0 | 0.26 |
| Weasel | 2022 | Mid-story canopy | 2 | 163.79* | 0.96 | 0.16 |
| Weasel | 2022 | Ground canopy | 3 | 164.62* | 1.8 | 0.11 |
| Weasel | 2022 | Riparian | 4 | 164.65* | 1.83 | 0.1 |
| Weasel | 2022 | Coarse woody debris | 5 | 165.01* | 2.19 | 0.09 |
| Weasel | 2022 | Snow depth | 6 | 165.07* | 2.25 | 0.08 |
| Weasel | 2022 | Ground- to mid- story structure | 7 | 165.91* | 3.09 | 0.06 |
| Weasel | 2022 | Snow depth and distance to riparian | 8 | 166.94* | 4.12 | 0.03 |
| Weasel | 2022 | Snow depth and coarse woody debris | 9 | 167.28* | 4.46 | 0.03 |
| Weasel | 2022 | Mature stand canopy cover | 10 | 167.32* | 4.5 | 0.03 |
| Weasel | 2022 | Riparian + structure | 11 | 167.93* | 5.11 | 0.02 |
| Weasel | 2022 | Type of riparian | 12 | 169.12* | 6.3 | 0.01 |
| Weasel | 2022 | Big tree characteristics | 13 | 169.5* | 6.68 | 0.01 |
| Weasel | 2022 | Vertical structure | 14 | 170.51* | 7.69 | 0.01 |
| Weasel | 2022 | Full habitat model | 15 | 172.84* | 10.02 | 0 |
| Mink | 2015 | Riparian | 1 | 149.51 | 0 | 0.55 |
| Mink | 2015 | Type of riparian | 2 | 150.28 | 0.77 | 0.37 |
| Mink | 2015 | Riparian + structure | 3 | 153.9 | 4.39 | 0.06 |
| Mink | 2015 | Null | 4 | 158.31 | 8.8 | 0.01 |
| Mink | 2015 | Ground canopy | 5 | 160.01 | 10.5 | 0 |
| Mink | 2015 | Mid-story canopy | 6 | 160.13 | 10.63 | 0 |
| Mink | 2015 | Coarse woody debris | 7 | 160.19 | 10.69 | 0 |
| Mink | 2015 | Big tree characteristics | 8 | 161.43 | 11.92 | 0 |
| Mink | 2015 | Ground- to mid- story structure | 9 | 161.76 | 12.25 | 0 |
| Mink | 2015 | Mature stand canopy cover | 10 | 162.06 | 12.55 | 0 |
| Mink | 2015 | Vertical structure | 11 | 166.14 | 16.63 | 0 |
| Mink | 2016 | Mid-story canopy | 1 | 114.38 | 0 | 0.42 |
| Mink | 2016 | Vertical structure | 2 | 115.71 | 1.33 | 0.21 |
| Mink | 2016 | Ground- to mid- story structure | 3 | 116.67 | 2.28 | 0.13 |
| Mink | 2016 | Big tree characteristics | 4 | 118.44 | 4.05 | 0.05 |
| Mink | 2016 | Riparian + structure | 5 | 118.68 | 4.29 | 0.05 |
| Mink | 2016 | Type of riparian | 6 | 119.53 | 5.15 | 0.03 |
| Mink | 2016 | Null | 7 | 119.81 | 5.42 | 0.03 |
| Mink | 2016 | Full habitat model | 8 | 120.17 | 5.79 | 0.02 |
| Mink | 2016 | Riparian | 9 | 120.38 | 6 | 0.02 |
| Mink | 2016 | Coarse woody debris | 10 | 120.76 | 6.38 | 0.02 |
| Mink | 2016 | Ground canopy | 11 | 121.95 | 7.57 | 0.01 |
| Mink | 2016 | Mature stand canopy cover | 12 | 123.1 | 8.72 | 0.01 |
| Mink | 2020 | Riparian | 1 | 86.75 | 0 | 0.37 |
| Mink | 2020 | Null | 2 | 86.78 | 0.02 | 0.37 |
| Mink | 2020 | Ground canopy | 3 | 88.73 | 1.98 | 0.14 |
| Mink | 2020 | Big tree characteristics | 4 | 89.11 | 2.35 | 0.12 |
| Mink | 2021 | Riparian | 1 | 41.24 | 0 | 0.49 |
| Mink | 2021 | Type of riparian | 2 | 43.37 | 2.13 | 0.17 |
| Mink | 2021 | Null | 3 | 43.83 | 2.59 | 0.13 |
| Mink | 2021 | Ground- to mid- story structure | 4 | 45.34 | 4.1 | 0.06 |
| Mink | 2021 | Ground canopy | 5 | 45.92 | 4.68 | 0.05 |
| Mink | 2021 | Snow depth | 6 | 46.01 | 4.77 | 0.05 |
| Mink | 2021 | Snow depth and distance to riparian | 7 | 47.87 | 6.63 | 0.02 |
| Mink | 2021 | Mature stand canopy cover | 8 | 48.12 | 6.88 | 0.02 |
| Mink | 2021 | Vertical structure | 9 | 49.16 | 7.91 | 0.01 |
| Mink | 2021 | Riparian + structure | 10 | 50.61 | 9.37 | 0 |
| Mink | 2022 | Riparian | 1 | 138.11 | 0 | 0.31 |
| Mink | 2022 | Type of riparian | 2 | 138.36 | 0.25 | 0.27 |
| Mink | 2022 | Snow depth and distance to riparian | 3 | 138.39 | 0.28 | 0.27 |
| Mink | 2022 | Riparian + structure | 4 | 139.77 | 1.66 | 0.14 |
| Mink | 2022 | Full habitat model | 5 | 146.07 | 7.96 | 0.01 |
| Mink | 2022 | Mid-story canopy | 6 | 149.49 | 11.38 | 0 |
| Mink | 2022 | Snow depth | 7 | 149.85 | 11.74 | 0 |
| Mink | 2022 | Ground- to mid- story structure | 8 | 151.58 | 13.47 | 0 |
| Mink | 2022 | Snow depth and coarse woody debris | 9 | 151.68 | 13.57 | 0 |
| Mink | 2022 | Null | 10 | 151.91 | 13.8 | 0 |
| Mink | 2022 | Vertical structure | 11 | 152.12 | 14.01 | 0 |
| Mink | 2022 | Big tree characteristics | 12 | 152.22 | 14.11 | 0 |
| Mink | 2022 | Coarse woody debris | 13 | 153.05 | 14.94 | 0 |
| Mink | 2022 | Ground canopy | 14 | 154.1 | 15.99 | 0 |
| Mink | 2022 | Mature stand canopy cover | 15 | 155.3 | 17.19 | 0 |
| Marten | 2015 | Ground canopy | 1 | 84.53* | 0 | 0.22 |
| Marten | 2015 | Null | 2 | 84.95* | 0.42 | 0.18 |
| Marten | 2015 | Mid-story canopy | 3 | 85.15* | 0.61 | 0.16 |
| Marten | 2015 | Ground- to mid- story structure | 4 | 85.41* | 0.88 | 0.14 |
| Marten | 2015 | Coarse woody debris | 5 | 86.7* | 2.17 | 0.07 |
| Marten | 2015 | Riparian | 6 | 87.21* | 2.68 | 0.06 |
| Marten | 2015 | Riparian + structure | 7 | 87.71* | 3.17 | 0.05 |
| Marten | 2015 | Mature stand canopy cover | 8 | 88.07* | 3.54 | 0.04 |
| Marten | 2015 | Big tree characteristics | 9 | 88.11* | 3.58 | 0.04 |
| Marten | 2015 | Type of riparian | 10 | 89* | 4.47 | 0.02 |
| Marten | 2015 | Vertical structure | 11 | 89.39* | 4.85 | 0.02 |
| Marten | 2015 | Full habitat model | 12 | 95.8* | 11.27 | 0 |
| Marten | 2016 | Mid-story canopy | 1 | 166.68* | 0 | 0.5 |
| Marten | 2016 | Ground- to mid- story structure | 2 | 167.95* | 1.28 | 0.27 |
| Marten | 2016 | Riparian + structure | 3 | 170.36* | 3.68 | 0.08 |
| Marten | 2016 | Ground canopy | 4 | 171.64* | 4.96 | 0.04 |
| Marten | 2016 | Null | 5 | 171.85* | 5.18 | 0.04 |
| Marten | 2016 | Vertical structure | 6 | 172.01* | 5.34 | 0.03 |
| Marten | 2016 | Coarse woody debris | 7 | 173.87* | 7.2 | 0.01 |
| Marten | 2016 | Riparian | 8 | 174.11* | 7.44 | 0.01 |
| Marten | 2016 | Mature stand canopy cover | 9 | 175.92* | 9.24 | 0 |
| Marten | 2016 | Big tree characteristics | 10 | 177.01* | 10.34 | 0 |
| Marten | 2016 | Type of riparian | 11 | 177.36* | 10.68 | 0 |
| Marten | 2016 | Full habitat model | 12 | 179.09* | 12.42 | 0 |
| Marten | 2020 | Coarse woody debris | 1 | 174.3* | 0 | 0.2 |
| Marten | 2020 | Null | 2 | 174.35* | 0.05 | 0.2 |
| Marten | 2020 | Mature stand canopy cover | 3 | 175.54* | 1.24 | 0.11 |
| Marten | 2020 | Mid-story canopy | 4 | 175.84* | 1.55 | 0.09 |
| Marten | 2020 | Snow depth | 5 | 176.08* | 1.78 | 0.08 |
| Marten | 2020 | Snow depth and coarse woody debris | 6 | 176.29* | 1.99 | 0.08 |
| Marten | 2020 | Ground canopy | 7 | 176.56* | 2.26 | 0.07 |
| Marten | 2020 | Riparian | 8 | 176.61* | 2.32 | 0.06 |
| Marten | 2020 | Vertical structure | 9 | 177.18* | 2.89 | 0.05 |
| Marten | 2020 | Ground- to mid- story structure | 10 | 178.24* | 3.94 | 0.03 |
| Marten | 2020 | Big tree characteristics | 11 | 179.91* | 5.61 | 0.01 |
| Marten | 2020 | Riparian + structure | 12 | 180.66* | 6.36 | 0.01 |
| Marten | 2020 | Type of riparian | 13 | 181.54* | 7.24 | 0.01 |
| Marten | 2020 | Full habitat model | 14 | 183.12* | 8.82 | 0 |
| Marten | 2021 | Riparian | 1 | 91.56* | 0 | 0.42 |
| Marten | 2021 | Type of riparian | 2 | 93.62* | 2.06 | 0.15 |
| Marten | 2021 | Snow depth and distance to riparian | 3 | 93.97* | 2.41 | 0.13 |
| Marten | 2021 | Ground canopy | 4 | 95.06* | 3.49 | 0.07 |
| Marten | 2021 | Coarse woody debris | 5 | 95.11* | 3.55 | 0.07 |
| Marten | 2021 | Mature stand canopy cover | 6 | 96.71* | 5.15 | 0.03 |
| Marten | 2021 | Vertical structure | 7 | 97.08* | 5.52 | 0.03 |
| Marten | 2021 | Null | 8 | 97.19* | 5.63 | 0.03 |
| Marten | 2021 | Ground- to mid- story structure | 9 | 97.42* | 5.85 | 0.02 |
| Marten | 2021 | Snow depth and coarse woody debris | 10 | 97.52* | 5.96 | 0.02 |
| Marten | 2021 | Full habitat model | 11 | 98.57* | 7 | 0.01 |
| Marten | 2021 | Snow depth | 12 | 99.31* | 7.75 | 0.01 |
| Marten | 2021 | Mid-story canopy | 13 | 99.48* | 7.92 | 0.01 |
| Marten | 2021 | Big tree characteristics | 14 | 102.25* | 10.69 | 0 |
| Marten | 2022 | Mid-story canopy | 1 | 166.39* | 0 | 0.21 |
| Marten | 2022 | Ground- to mid- story structure | 2 | 166.87* | 0.48 | 0.17 |
| Marten | 2022 | Ground canopy | 3 | 167.13* | 0.74 | 0.14 |
| Marten | 2022 | Null | 4 | 167.97* | 1.58 | 0.1 |
| Marten | 2022 | Riparian + structure | 5 | 168.18* | 1.79 | 0.09 |
| Marten | 2022 | Riparian | 6 | 168.9* | 2.51 | 0.06 |
| Marten | 2022 | Vertical structure | 7 | 169.59* | 3.2 | 0.04 |
| Marten | 2022 | Coarse woody debris | 8 | 169.7* | 3.31 | 0.04 |
| Marten | 2022 | Snow depth | 9 | 169.73* | 3.34 | 0.04 |
| Marten | 2022 | Mature stand canopy cover | 10 | 170.44* | 4.05 | 0.03 |
| Marten | 2022 | Big tree characteristics | 11 | 170.72* | 4.33 | 0.02 |
| Marten | 2022 | Type of riparian | 12 | 170.77* | 4.38 | 0.02 |
| Marten | 2022 | Snow depth and distance to riparian | 13 | 170.84* | 4.45 | 0.02 |
| Marten | 2022 | Snow depth and coarse woody debris | 14 | 171.38* | 4.99 | 0.02 |
| Marten | 2022 | Full habitat model | 15 | 175.22* | 8.83 | 0 |
| Fisher | 2020 | Snow depth | 1 | 166.57 | 0 | 0.21 |
| Fisher | 2020 | Null | 2 | 166.83 | 0.26 | 0.18 |
| Fisher | 2020 | Snow depth and distance to riparian | 3 | 167.48 | 0.91 | 0.13 |
| Fisher | 2020 | Snow depth and coarse woody debris | 4 | 168.39 | 1.82 | 0.08 |
| Fisher | 2020 | Riparian | 5 | 168.48 | 1.91 | 0.08 |
| Fisher | 2020 | Coarse woody debris | 6 | 168.56 | 1.99 | 0.08 |
| Fisher | 2020 | Ground canopy | 7 | 168.64 | 2.07 | 0.07 |
| Fisher | 2020 | Mid-story canopy | 8 | 168.88 | 2.31 | 0.06 |
| Fisher | 2020 | Mature stand canopy cover | 9 | 169.79 | 3.21 | 0.04 |
| Fisher | 2020 | Ground- to mid- story structure | 10 | 170.85 | 4.28 | 0.02 |
| Fisher | 2020 | Big tree characteristics | 11 | 171.41 | 4.84 | 0.02 |
| Fisher | 2020 | Type of riparian | 12 | 172.28 | 5.7 | 0.01 |
| Fisher | 2020 | Riparian + structure | 13 | 172.86 | 6.29 | 0.01 |
| Fisher | 2020 | Vertical structure | 14 | 173.94 | 7.36 | 0.01 |
| Fisher | 2020 | Full habitat model | 15 | 180.25 | 13.68 | 0 |
| Fisher | 2021 | Riparian | 1 | 128.3 | 0 | 0.43 |
| Fisher | 2021 | Type of riparian | 2 | 129.18 | 0.88 | 0.28 |
| Fisher | 2021 | Full habitat model | 3 | 131.65 | 3.35 | 0.08 |
| Fisher | 2021 | Vertical structure | 4 | 132.78 | 4.49 | 0.05 |
| Fisher | 2021 | Ground canopy | 5 | 132.86 | 4.56 | 0.04 |
| Fisher | 2021 | Coarse woody debris | 6 | 132.93 | 4.63 | 0.04 |
| Fisher | 2021 | Mature stand canopy cover | 7 | 134.13 | 5.83 | 0.02 |
| Fisher | 2021 | Snow depth and distance to riparian | 8 | 134.24 | 5.94 | 0.02 |
| Fisher | 2021 | Ground- to mid- story structure | 9 | 135.05 | 6.75 | 0.01 |
| Fisher | 2021 | Snow depth and coarse woody debris | 10 | 135.18 | 6.88 | 0.01 |
| Fisher | 2021 | Null | 11 | 136.48 | 8.18 | 0.01 |
| Fisher | 2021 | Snow depth | 12 | 138.38 | 10.08 | 0 |
| Fisher | 2021 | Mid-story canopy | 13 | 138.61 | 10.31 | 0 |
| Fisher | 2021 | Riparian + structure | 14 | 140.41 | 12.11 | 0 |
| Fisher | 2022 | Snow depth and distance to riparian | 1 | 154.22 | 0 | 0.21 |
| Fisher | 2022 | Null | 2 | 154.46 | 0.24 | 0.19 |
| Fisher | 2022 | Snow depth | 3 | 155.4 | 1.18 | 0.12 |
| Fisher | 2022 | Riparian | 4 | 155.42 | 1.2 | 0.12 |
| Fisher | 2022 | Coarse woody debris | 5 | 155.78 | 1.56 | 0.1 |
| Fisher | 2022 | Mid-story canopy | 6 | 156.66 | 2.44 | 0.06 |
| Fisher | 2022 | Ground canopy | 7 | 156.66 | 2.44 | 0.06 |
| Fisher | 2022 | Snow depth and coarse woody debris | 8 | 157.27 | 3.05 | 0.05 |
| Fisher | 2022 | Mature stand canopy cover | 9 | 157.88 | 3.66 | 0.03 |
| Fisher | 2022 | Ground- to mid- story structure | 10 | 158.92 | 4.7 | 0.02 |
| Fisher | 2022 | Type of riparian | 11 | 159.78 | 5.56 | 0.01 |
| Fisher | 2022 | Riparian + structure | 12 | 159.94 | 5.72 | 0.01 |
| Fisher | 2022 | Big tree characteristics | 13 | 160.68 | 6.46 | 0.01 |
| Fisher | 2022 | Vertical structure | 14 | 162.17 | 7.95 | 0 |
| Fisher | 2022 | Full habitat model | 15 | 168.36 | 14.14 | 0 |
