# Supplementary material for: Riparian Areas and Fine‐Scale Forest Cover and Structure Drive Occupancy Patterns of Sympatric Mustelids
Source: Ecol Evol. 2025 May 7;15(5):e71370. doi: 10.1002/ece3.71370 (PMC12058454; doi:10.1002/ece3.71370)
Supplement: Supplementary file 2 — Table S2: Multi‐species occupancy models ranked by AICc for fisher ( Pekania pennanti ), American marten ( Martes americana ), American mink (Neogale vison), and short‐tailed weasel ( Mustela erminea ) in 2015–2016 and 2020–2022 in the John Prince Research Forest, British Columbia, Canada. Fit 1 describes an intercept only model, assuming independence between species, and fit 2 describes an intercept‐only model that assumes dependence between species. Both fit 1 and fit 2 are considered null models for occupancy. [file ECE3-15-e71370-s001.docx]

# **Table S2 –** Multi-species occupancy models ranked by AIC*c* for fisher (*Pekania pennanti*), American marten (*Martes americana*), American mink (*Neogale vison*), and short-tailed weasel (*Mustela erminea*) in 2015-2016 and 2020-2022 in the John Prince Research Forest, British Columbia, Canada. Fit 1 describes an intercept only model, assuming independence between species, and fit 2 describes an intercept-only model that assumes dependence between species. Both fit 1 and fit 2 are considered null models for occupancy.

| Year | Species | model | rank | K | AICc | ΔAICc | AICc ω*_i_* |
| --- | --- | --- | --- | --- | --- | --- | --- |
| 2015-2016 | Marten + Weasel | ρ(.)ρ(.) ψ(cc3-10) | 1 | 8 | 854.82 | 0 | 1 |
| 2015-2016 | Marten + Weasel | ρ(.)ρ(.) ψ(.) (fit 1) | 2 | 4 | 868.4 | 13.59 | 0 |
| 2015-2016 | Marten + Weasel | ρ(.)ρ(.) ψ(.) (fit 2) | 3 | 5 | 870.28 | 15.46 | 0 |
| 2015-2016 | Marten + Weasel | ρ(.)ρ(.) ψ(distance_to_riparian) | 4 | 8 | 875.32 | 20.51 | 0 |
| 2015-2016 | Marten + Weasel | ρ(.)ρ(.) ψ(cc0-3) | 5 | 10 | 1080.73 | 225.91 | 0 |
| 2015-2016 | Marten + Weasel | ρ(.)ρ(.) ψ(cc3-10+cc0-3) | 6 | 13 | 1088.49 | 233.68 | 0 |
| 2020-2022 | Marten + Weasel | ρ(.)ρ(.) ψ(cc3-10) | 1 | 10 | 1771.12 | 0 | 1 |
| 2020-2022 | Marten + Weasel | ρ(.)ρ(.) ψ(snow_depth) | 2 | 10 | 1784.17 | 13.05 | 0 |
| 2020-2022 | Marten + Weasel | ρ(.)ρ(.) ψ(cc10) | 3 | 10 | 1785.41 | 14.3 | 0 |
| 2020-2022 | Marten + Weasel | ρ(.)ρ(.) ψ(cc10+cc0-3) | 4 | 13 | 1793.13 | 22.01 | 0 |
| 2020-2022 | Marten + Weasel | ρ(.)ρ(.) ψ(distance_to_riparian) | 5 | 10 | 1819.12 | 48 | 0 |
| 2020-2022 | Marten + Weasel | ρ(.)ρ(.) ψ(.) (fit 1) | 6 | 4 | 1835.54 | 64.42 | 0 |
| 2020-2022 | Marten + Weasel | ρ(.)ρ(.) ψ(.) (fit 2) | 7 | 5 | 1837.81 | 66.69 | 0 |
| 2015-2016 | Marten + Mink | ρ(.)ρ(.) ψ(cc3-10) | 1 | 8 | 819.11 | 0 | 0.81 |
| 2015-2016 | Marten + Mink | ρ(.)ρ(.) ψ(cc0-3) | 2 | 8 | 822.13 | 3.02 | 0.18 |
| 2015-2016 | Marten + Mink | ρ(.)ρ(.) ψ(cc10+cc0-3) | 3 | 11 | 828.37 | 9.26 | 0.01 |
| 2015-2016 | Marten + Mink | ρ(.)ρ(.) ψ(.) (fit 1) | 4 | 5 | 830.39 | 11.27 | 0 |
| 2015-2016 | Marten + Mink | ρ(.)ρ(.) ψ(.) (fit 2) | 5 | 4 | 830.83 | 11.72 | 0 |
| 2015-2016 | Marten + Mink | ρ(.)ρ(.) ψ(distance_to_riparian) | 6 | 8 | 831.65 | 12.53 | 0 |
| 2015-2016 | Marten + Mink | ρ(.)ρ(.) ψ(cc10) | 7 | 8 | 833.74 | 14.63 | 0 |
| 2020-2022 | Marten + Mink | ρ(.)ρ(.) ψ(cc3-10) | 1 | 8 | 1164.73 | 0 | 0.5 |
| 2020-2022 | Marten + Mink | ρ(.)ρ(.) ψ(distance_to_riparian) | 2 | 8 | 1165.03 | 0.29 | 0.43 |
| 2020-2022 | Marten + Mink | ρ(.)ρ(.) ψ(.) (fit 1) | 3 | 4 | 1169.13 | 4.4 | 0.06 |
| 2020-2022 | Marten + Mink | ρ(.)ρ(.) ψ(.) (fit 2) | 4 | 5 | 1171.31 | 6.58 | 0.02 |
| 2015-2016 | Mink + Weasel | ρ(.)ρ(.) ψ(cc3-10) | 1 | 8 | 594.82 | 0 | 0.64 |
| 2015-2016 | Mink + Weasel | ρ(.)ρ(.) ψ(.) (fit 1) | 2 | 4 | 597.6 | 2.78 | 0.16 |
| 2015-2016 | Mink + Weasel | ρ(.)ρ(.) ψ(distance_to_riparian) | 3 | 8 | 598.02 | 3.19 | 0.13 |
| 2015-2016 | Mink + Weasel | ρ(.)ρ(.) ψ(.) (fit 2) | 4 | 5 | 599.1 | 4.28 | 0.08 |
| 2015-2016 | Mink + Weasel | ρ(.)ρ(.) ψ(cc0-3) | 5 | 10 | 1080.73 | 485.9 | 0 |
| 2020-2022 | Mink + Weasel | ρ(.)ρ(.) ψ(distance_to_riparian) | 1 | 10 | 1173.89 | 0 | 0.99 |
| 2020-2022 | Mink + Weasel | ρ(.)ρ(.) ψ(cc3-10) | 2 | 10 | 1184.16 | 10.27 | 0.01 |
| 2020-2022 | Mink + Weasel | ρ(.)ρ(.) ψ(snow_depth) | 3 | 10 | 1186.71 | 12.82 | 0 |
| 2020-2022 | Mink + Weasel | ρ(.)ρ(.) ψ(cc10) | 4 | 10 | 1187.32 | 13.43 | 0 |
| 2020-2022 | Mink + Weasel | ρ(.)ρ(.) ψ(cc10+cc0-3) | 5 | 13 | 1193.73 | 19.83 | 0 |
| 2020-2022 | Mink + Weasel | ρ(.)ρ(.) ψ(.) (fit 1) | 6 | 4 | 1225.77 | 51.88 | 0 |
| 2020-2022 | Mink + Weasel | ρ(.)ρ(.) ψ(.) (fit 2) | 7 | 5 | 1226.44 | 52.55 | 0 |
| 2020-2022 | Fisher + Mink | ρ(.)ρ(.) ψ(distance_to_riparian) | 1 | 8 | 717.68 | 0 | 0.99 |
| 2020-2022 | Fisher + Mink | ρ(.)ρ(.) ψ(.) (fit 1) | 2 | 4 | 727.48 | 9.81 | 0.01 |
| 2020-2022 | Fisher + Mink | ρ(.)ρ(.) ψ(.) (fit 2) | 3 | 5 | 729.82 | 12.14 | 0 |
| 2020-2022 | Fisher + Mink | ρ(.)ρ(.) ψ(cc3-10) | 4 | 8 | 736.24 | 18.56 | 0 |
| 2020-2022 | Fisher + Marten | ρ(.)ρ(.) ψ(cc3-10) | 1 | 9 | 1316.76 | 0 | 0.94 |
| 2020-2022 | Fisher + Marten | ρ(.)ρ(.) ψ(snow_depth) | 2 | 9 | 1323.79 | 7.04 | 0.03 |
| 2020-2022 | Fisher + Marten | ρ(.)ρ(.) ψ(cc0-3) | 3 | 9 | 1324.84 | 8.08 | 0.02 |
| 2020-2022 | Fisher + Marten | ρ(.)ρ(.) ψ(cc10+cc3-10+cc0-3) | 4 | 15 | 1326.32 | 9.56 | 0.01 |
| 2020-2022 | Fisher + Marten | ρ(.)ρ(.) ψ(distance_to_riparian) | 5 | 9 | 1327.17 | 10.41 | 0.01 |
| 2020-2022 | Fisher + Marten | ρ(.)ρ(.) ψ(cc10) | 6 | 9 | 1328.45 | 11.69 | 0 |
| 2020-2022 | Fisher + Marten | ρ(.)ρ(.) ψ(.) (fit 2) | 7 | 5 | 1331.91 | 15.15 | 0 |
| 2020-2022 | Fisher + Marten | ρ(.)ρ(.) ψ(.) (fit 1) | 8 | 4 | 1337.25 | 20.5 | 0 |
| 2020-2022 | Fisher + Weasel | ρ(.)ρ(.) ψ(cc10) | 1 | 9 | 1351.93 | 0 | 0.99 |
| 2020-2022 | Fisher + Weasel | ρ(.)ρ(.) ψ(distance_to_riparian) | 2 | 9 | 1360.59 | 8.66 | 0.01 |
| 2020-2022 | Fisher + Weasel | ρ(.)ρ(.) ψ(.) (fit 1) | 3 | 4 | 1393.89 | 41.96 | 0 |
| 2020-2022 | Fisher + Weasel | ρ(.)ρ(.) ψ(.) (fit 2) | 4 | 5 | 1396.09 | 44.16 | 0 |
| 2020-2022 | Fisher + Weasel | ρ(.)ρ(.) ψ(cc3-10) | 5 | 8 | 1398.6 | 46.67 | 0 |
